# A correlation coefficient-based feature selection approach for virus-host protein-protein interaction prediction

**DOI:** 10.1371/journal.pone.0285168

**Published:** 2023-05-02

**Authors:** Ahmed Hassan Ibrahim, Onur Can Karabulut, Betül Asiye Karpuzcu, Erdem Türk, Barış Ethem Süzek

**Affiliations:** 1 Bioinformatics Graduate Program, Graduate School of Natural and Applied Sciences, Muğla Sıtkı Koçman University, Muğla, Turkey; 2 Department of Computer Engineering, Faculty of Engineering, Muğla Sıtkı Koçman University, Muğla, Turkey; 3 Georgetown University Medical Center, Biochemistry and Molecular & Cellular Biology, Washington DC, United States of America; Kingston University, UNITED KINGDOM

## Abstract

Prediction of virus-host protein-protein interactions (PPI) is a broad research area where various machine-learning-based classifiers are developed. Transforming biological data into machine-usable features is a preliminary step in constructing these virus-host PPI prediction tools. In this study, we have adopted a virus-host PPI dataset and a reduced amino acids alphabet to create tripeptide features and introduced a correlation coefficient-based feature selection. We applied feature selection across several correlation coefficient metrics and statistically tested their relevance in a structural context. We compared the performance of feature-selection models against that of the baseline virus-host PPI prediction models created using different classification algorithms without the feature selection. We also tested the performance of these baseline models against the previously available tools to ensure their predictive power is acceptable. Here, the Pearson coefficient provides the best performance with respect to the baseline model as measured by AUPR; a drop of 0.003 in AUPR while achieving a 73.3% (from 686 to 183) reduction in the number of tripeptides features for random forest. The results suggest our correlation coefficient-based feature selection approach, while decreasing the computation time and space complexity, has a limited impact on the prediction performance of virus-host PPI prediction tools.

## Introduction

Viruses are among the most common causes of infectious diseases worldwide leading to a substantial burden on human health and the global economy. The complex set of virus-host cell interactions comprises the initial recognition and binding of the virion to the host, cellular entry, dissemination, and finally a productive or a latent infection; all of which need to be elucidated for a comprehensive understanding of viral diseases. To this end, the protein-protein interactions (PPIs), occurring as the first physical contact between the viral protein and the host receptor(s), have been a hot topic of investigation in medical, biological, and *in silico* research [1, 2].

Taking the cost and labor intensiveness of wet-lab techniques to assess PPIs, efforts have been driven towards computational methods including machine-learning algorithms for intra-species and inter-species PPI prediction. However, tools developed specifically for predicting intra-species PPIs cannot effectively distinguish interactions taking place between proteins of an organism from those taking place between the proteins of that organism and a pathogen [3]. For this reason, these general PPI predictors are not appropriate for inter-species PPIs which involves an additional difficulty [4]. Briefly, the prediction of PPIs between a virus and host is different from the prediction of PPIs within the same organism primarily due to the pace of evolution and conservation of interacting counterparts. Viruses tend to evolve at a higher rate and a peculiarity of viral domains is their tendency to evolve by convergence, mimicking host interfaces and allowing their proteins to target and compete for host factors usually involved in crucial cellular processes [5].

There is an armamentarium of computational methods for PPI identification. These include approaches based on protein co-evolution [6], sequence similarity, and domain-domain interaction patterns devised to, for example, predict genome-scale host-pathogen PPIs [7], structural annotation and modeling [8] and self-adjustable Gaussian Network Model to determine binding pockets for small peptides or molecules, which is particularly useful in the discovery of PPI-inhibitory pharmaceutical compounds [9]. Among the computational methods, machine learning-based virus-host PPI prediction approaches handle PPI identification as a binary classification problem. Such a machine-learning approach involves first collecting a set of known positive (interacting) and negative (non-interacting) protein pairs in order to construct training and test dataset(s). Next, a feature vector is gathered from such PPI samples for which a plethora of feature extraction techniques have been developed including structure-based [10–12], sequence-based [3, 13, 14], and domain-based [11, 15, 16] techniques. There are also techniques that consider ontology [17, 18], gene expression [19], and evolutionary profiles [20, 21] of proteins. Eventually, the feature vector serves during the training and testing of machine-learning-based virus-host PPI prediction models to distinguish between positive and negative PPIs.

Several machine-learning-based virus-host PPI prediction tools have been previously documented and used different algorithms such as support vector machines (SVM) [3, 22, 23], random forest (RF) [24], and gradient boosting machine (XGBoost) [13, 25].

In the SVM-based model, called DeNovo, amino acid sequence similarity-based features have been used [3]. The authors used a feature extraction scheme originally developed by Shen et al. [23]. This scheme incorporates clustering of amino acids, uses clusters to encode residues, and calculates the frequencies of such encoded residues in triplets, also called tripeptides. DeNovo also employs a sequence similarity-based strategy for sampling the negative virus-host PPI data set for SVM training. The XGBoost classifier named HOPITOR [13] also uses the negative sampling strategy described by DeNovo and, similarly, relies on the feature extraction scheme of Shen et al. [23].

SVM-based tool VirusHostPPI [4] also applies the same feature extraction scheme and incorporated the relative frequency of amino acid triplets (RFAT) constituting 686 elements for each pair of host and virus proteins into their feature vector which was supplemented by further aspects of protein sequence-based features: the frequency difference of amino acid triplets (FDAT) between virus and host proteins; amino acid composition (AC) in each pair of host and virus proteins; as well as composition, transition and distribution of amino acid groups as explained in the study of You and colleagues [26]. In the RF-based classifier InterSPPI-HVPPI [24], the protein sequences were embedded using the doc2vec model where a corpus of sequence information is used for training a model to compute protein sequence-specific features.

When developing a machine-learning-based classification model, the process of selecting a subset of original features, i.e. feature selection, is used to reduce the dataset by removing irrelevant and redundant features, leading to improved data quality. While reducing computation time and space complexity, feature selection potentially increases the accuracy of models [27].

Although several virus-host PPI prediction tools use tripeptide frequencies as features, none of them considered whether it is possible to achieve similar or better performances with smaller feature vector sizes. Here, we applied correlation coefficient thresholds to glean the tripeptides to effectively select a minimal set of features for PPI prediction. Correlation was previously used in *in silico* research. Such that, the correlation between protein sequences to determine interaction partners [28], and between protein domains to capture co-evolution signals in predicting intra-species PPIs [29].

In this study, we 1) adapted a previously curated virus-host PPI dataset; 2) extracted features depending on normalized tripeptide frequencies based on the 7-letter reduced alphabet of amino acids proposed by Shen et al. [23] to create protein sequence-based feature vectors; 3) applied different correlation coefficient metrics with various thresholds to select subsets from such features; 4) built machine-learning-based PPI prediction models to assess the effectiveness of our feature selection approach to test whether it is still yielding a reasonable performance comparable to the previously available methods. Thus, we aim to highlight the value of feature selection which allows us to reduce the computation time and space complexity of virus-host PPI prediction.

## Materials and methods

### Dataset preparation

In this study, we used virus-host protein pairs compiled by Yang and colleagues, accessible through the official tool website of InterSPPI-HVPPI (http://zzdlab.com/hvppi/). For this dataset, they used the manually curated PPI data from Host-Pathogen Interaction Database (HPIDB; version 3.0) [30] to obtain the positive (interacting) host-pathogen protein pairs wherein 22,653 human-virus PPIs were selected after filtering out the redundant PPIs (based on sequence identity threshold <0.5), non-physical interactions, and any interactions involving a protein size of <30 or >5,000 amino acids. Further, they downloaded protein data available in SwissProt [31] and produced the negative data set using Dissimilarity Based Negative Sampling method. Eventually, their dataset has a positive to negative ratio of 1:10. The entire dataset was handled in 3 random partitions of equal size both for training and three test sets to reduce sampling bias each of which was further also split into 80% training and 20% test set.

**Fig 1:**
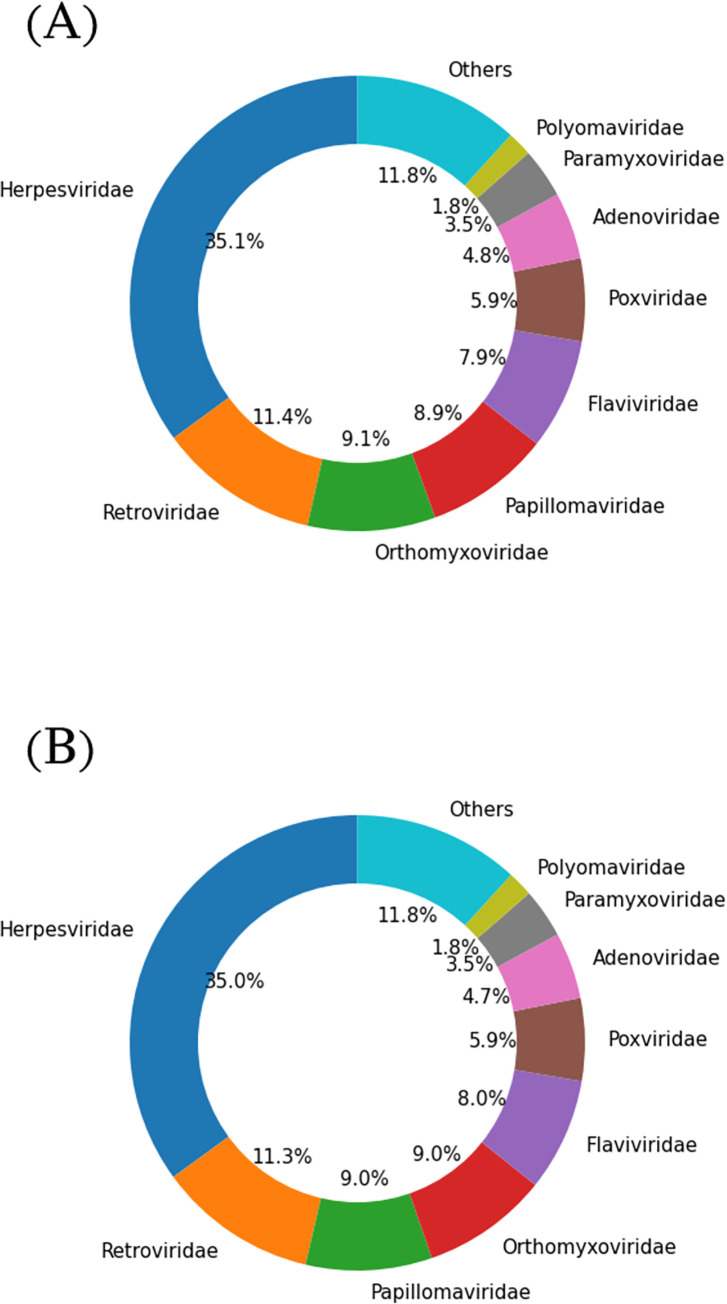
Distribution of viral families in the datasets. (A) shows the distribution of viral families in the training dataset and (B) shows the distribution of viral families in test datasets.

In our study, we combined these three random partitions of the training dataset and test dataset separately and removed duplicated records. Also, the records that intersect between training and test sets were removed from the training dataset. Eventually, we had a combined training set with 14,283 interacting (+) pairs and 262,731 non-interacting (-) pairs and a combined test set with 8,375 interacting (+) pairs and 114,563 non-interacting (-) pairs. As these source datasets only provide the accession identifiers, corresponding protein sequences were obtained from UniProtKB (https://www.uniprot.org/) [31]. This dataset represents proteins from a diverse set of viral taxa (n > 10) an illustration of which is depicted in Fig 1. In both training and test sets, *Herpesviridae* was the most prevalent family with a ratio of about 35%, and the distribution of families was comparable.

### Feature extraction for virus-host PPI prediction

In the available virus-host PPI prediction tools (DeNovo [3], HOPITOR [13], VirusHostPPI [4]), a common approach for extracting features was the use of the frequencies of amino acids in units of three adjacent residues (i.e. tripeptide) where each residue is encoded with a number. Due to its commonality, we have also utilized the same approach to extract protein-sequence-based features. Briefly, this feature extraction scheme described by Shen et al. [23] encompasses the following: first, 20 amino acids were divided into seven clusters encoded with numbers from 1 to 7, in the given order, from 1 to 7, in the given order, {*A, V, G}, {I, L,F, P}, {Y, M, T, S}, {H, N, Q, W}, {R, K}, {D, E}*, and *{C}* based on similarities of physicochemical properties known to drive most PPIs (dipoles and volumes of side chains). Amino acids in each protein of the PPI pair, one from the host and one from the virus, are mapped to the corresponding cluster numbers. Next, the frequency of each tripeptide is calculated in both virus and host proteins, generating a (7^3 = 343 dimensional) feature vector. The feature vectors are then normalized using the min-max approach over [0, 1] for each protein independently. These two normalized vectors of a protein pair (interacting or non-interacting) are concatenated into a single feature vector.

We adopted exactly the same feature extraction method as described above and thus for each of the protein sequences constituting a virus-host pair in our combined sets we ended up with a feature vector at a size of 2 × 343 features.

### Correlation-based feature selection

Here we utilized correlations between features which are the normalized frequencies of virus tripeptides on one side and host tripeptides on the other side. To select the most correlated features, we first generated a correlation coefficient matrix using one of the following correlation coefficient metrics; Pearson (PS), Spearman’s rank (SM), and Kendall’s τ (KT). In each one of these matrices, each virus-host protein pair -in the positive training dataset- is represented based on the respective feature vectors (i.e. normalized frequencies of all possible tripeptides). The correlation coefficient, though expected to be low due to taxa and protein diversity we included in the dataset, herein is used as a metric to identify the relation between interacting virus and their host peptides. The correlation coefficients were calculated using pearsonr, spearmanr, and kendalltau which are provided by the SciPy Python library [32].

**Fig 2:**
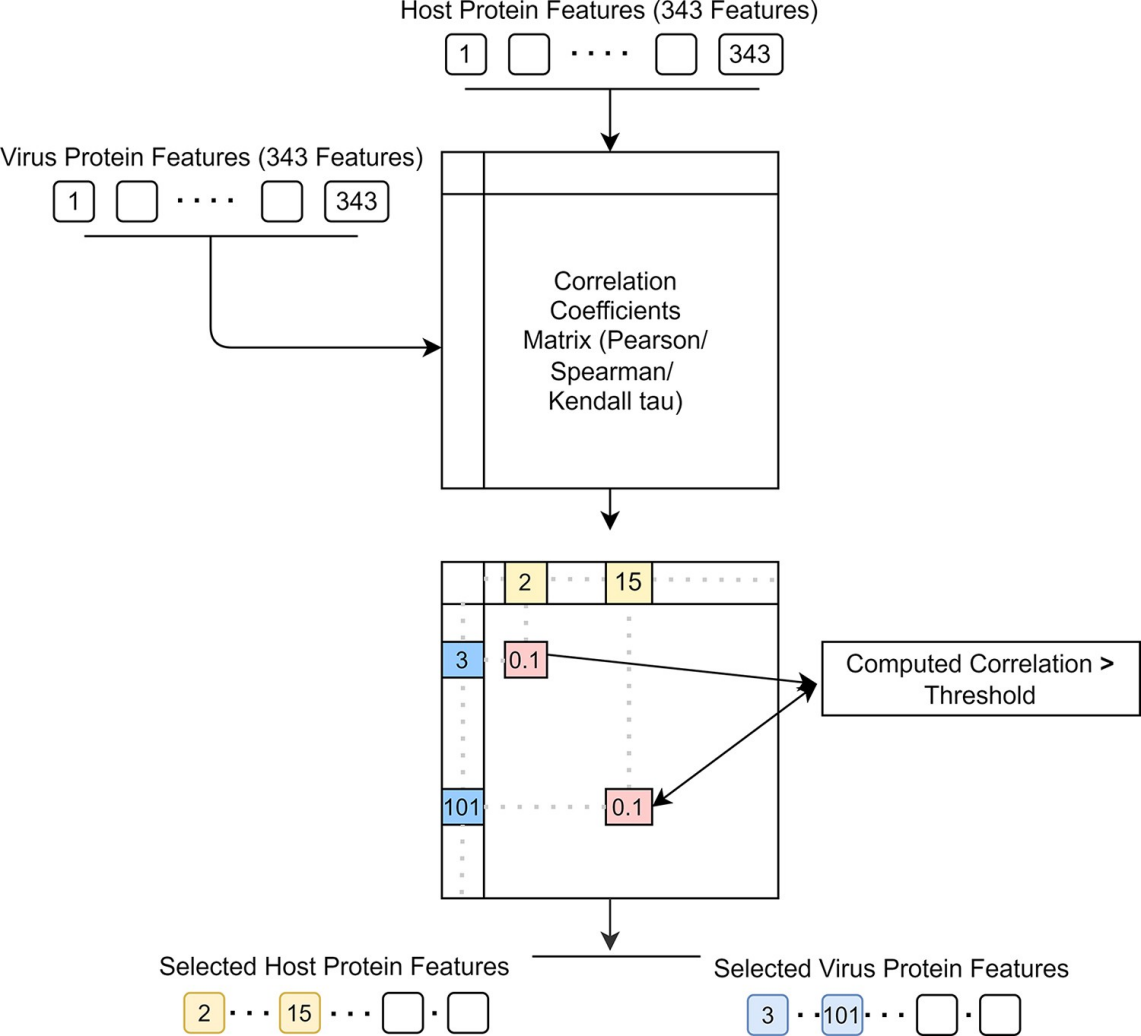
Overview of feature selection. Computation of correlated instances and selection based on correlation coefficient threshold is shown. In this case, host protein feature #2 (second tripeptide’s normalized frequency) and virus protein feature #3 has a computed correlation above the threshold. Likewise, host protein feature #15 and virus protein feature #101.

In each of the calculated correlation matrices, different thresholds were applied to filter out the correlating host and virus features starting from 0 with increments of 0.01 as long as at least one feature per virus or host protein is selected. At each correlated instance, (i.e. the correlation metric is above the threshold) the corresponding host feature and the corresponding viral feature, which stands for an encoded tripeptide, were included in the selected host feature set and the selected viral feature set, respectively. Features were added in a unique fashion. A depiction of the complete process of the feature selection is provided in Fig 2.

### Virus–host PPI prediction model construction

To measure the impact of feature selection on model performance, we first constructed baseline virus-host PPI prediction models based on random forest (RF), support vector machine (SVM), and multi-layer perceptron (MLP) algorithms using the full feature vector (686-dimensional) without any feature selection. For these models, we used default parameters of respective algorithms (for RF: n_estimators = 100, criterion = gini, and max_features = auto, for SVM: kernel = rbf, C = 1, and gamma = ‘scale’, for MLP: hidden_layer_sizes = (100,), activation = relu, solver = ‘adam’, alpha = 0.0001, and learning_rate = ‘constant’). In order to deal with the class imbalance towards the negative class in our dataset, we employed random undersampling during the training process of the models.

After that, using our feature selection approach described in the section entitled Correlation-Based Feature Selection, we developed different prediction models using these 3 machine-learning algorithms (RF, SVM, MLP) employing a distinct reduced set of features based on 3 different correlation coefficient metrics (PS, SM, KT) at different threshold levels. We implemented a 5-fold cross validation (CV) where each virus family taxon is proportionally represented in each validation fold instead of forming completely random validation folds where virus families are not necessarily represented proportionally. In other words, the stratification in cross validation not only factored in positive to negative ratio but also the representation of virus families in each fold. This CV strategy helped conservation of virus family specific tripeptide patterns those are critical to our feature selection approach. The results are presented in S1 Table. The prediction models have been implemented using the Scikit-Learn library [33] for the Python programming language.

### Performance evaluation metrics for virus-host PPI prediction models

To measure the impact of feature selection compared against the baseline, we used the following performance metrics: true positive rate (TPR), true negative rate (TNR), accuracy (ACC), F-score (F1), Matthew’s correlation coefficient (MCC), area under curve (AUC), and area under precision-recall curve (AUPR). These measures are defined as follows:

$$TPR=Sensitivity=Recall=1-FNR\;\frac{TP}{TP+FN}$$ (1)

$$\mathrm{TNR}=\mathrm{Specificity}=1-\mathrm{FPR}=\frac{TN}{TN+FP}$$ (2)

$$\mathrm{Precision}=\frac{TP}{TP+FP}$$ (3)

$$\mathrm{FDR}=1-\mathrm{PPV}=\frac{FP}{FP+TN}$$ (4)

$$\mathrm{Accuracy}=\frac{TP+TN}{TP+TN+FP+FN}$$ (5)

$$F‐\mathrm{Score}=\frac{2*Precision*Recall}{Precision+Recall}$$ (6)

$$AUC=\int_{x=0}^{1}TPR({FPR}^{-1}(x))dx$$ (7)

$$MCC=\frac{TP*TN-FP*FN}{\sqrt{\left(TP+FP)(TP+FN)(TN+FP)(TN+FN\right)}}$$ (8)

$$AUPR=\int_{x=0}^{1}Precision({Recall}^{-1}(x))dx$$ (9)

TP (true positive) is the number of anticipated positive PPIs that really interact. FP (false positive) is the number of expected positive PPIs that are really negative. TN (true negative) is the number of PPIs projected negatively that is actually negative, whereas FN (false negative) is the number of PPIs predicted negatively that are actually positive. Accuracy is the degree to which a measured value is near to the real (true) value. The F1 is a metric for determining how accurate a model is on a given dataset. It’s used to assess binary classification systems that divide examples into ’positive’ and ’negative’ classes. The area under the receiver operating characteristics (ROC) curve, also known as AUC, is one of the most essential assessment metrics for evaluating the effectiveness of any classification model. It indicates how well the model can discriminate between different classes where a value of 0.5 is equivalent to a predictive power of flip coin, and 1.0 stands for the highest achievable predictive power. MCC is a correlation coefficient between observed and expected outcomes that are used to assess the quality of binary classification. AUPR is an alternative to AUC particularly appropriate for evaluating the performance of models built on imbalanced datasets where a baseline value of P/(P+N) depends on the class distribution [34].

### Investigation of selected tripeptide features in virus–host PPI structural context

In order to make a comparison with the available protein structure data to help interpretation of our correlation-based feature selection, we have used the experimentally verified protein structure data retrieved from RSCB Protein Databank (PDB) [35]. First, we downloaded the PDB files containing a viral protein and a host protein together (interacting) from our positive training and test datasets. Thus, we downloaded 211 PDB files, illustrating both in conjunction, out of which organism attribution was lacking for viral or host protein chains in 22 files and only 130 had a reported resolution value. Out of these 130 structures, PDB ID:4YSI has the highest (resolution (app. 1.0 Å) and contains Kaposi sarcoma herpesvirus (KSHV) vIRF1 protein with human ubiquitin-specific protease 7 (USP7). We filtered the entries which have a resolution up to 3.0 Å. Thus, we ended up with 94 PDB entries. See S3 Table for a complete list of PDB entries.

For these entries, we used Bio.PDB.PDBParser.PDBParser module of Biopython package [36] in Python to figure out the protein-protein contacts applying a threshold of < 5 Å distance between the alpha carbon (Cα) atoms along the chains of peptides as described by Viloria et al. [37]. Accordingly, to identify such amino acids which are the most important for the interaction, for each protein structure file, we traversed all residues along the viral peptide chain using a sliding window (at a size of 3 amino acids) and calculated the distance from its Cα atom to the Cα atom of the host peptide chain. In this way, we picked up the PDB-based virus tripeptides. We did the same calculation traversing the host peptide chain and calculating the distance to the virus we picked up the PDB-based host tripeptides. Using the same encoding scheme based on the reduced 7-letter amino acid alphabet as we used while selecting our correlation-based set of features, we have also converted these tripeptides and hereafter referred to them as contact tripeptides.

We conducted a quantitative and a qualitative analysis of selected tripeptide features in the structural context of virus-host PPIs. For quantitative analysis of the selected tripeptide features and contact tripeptides derived from PDB structures, we first checked for their intersection. Here, the intersection infers those tripeptides co-occurring in both sets. We searched whether our feature selection is favoring contact tripeptides using Fisher’s exact test at a significance level of p < 0.05. Our null hypothesis is that the proportion of intersection is higher among the tripeptides selected by the correlation-based approach in comparison to the proportion of intersection among the non-selected tripeptides. The Fisher’s exact test was conducted using the stats module under SciPy Python library (version 1.9.3) in Python [32].

## Results and discussion

### Assessment of correlation-based feature selection on virus–host PPI prediction performance

We tested the performance of our PPI prediction models (RF, SMV, MLP) constructed without any feature selection (686 features) trained using a training set, and tested using the test set as described in Section Virus–host PPI Prediction Model Construction. The performances of our baseline models, as well as the performance of other available tools (DeNovo [3], HOPITOR [13], InterSPPI-HVPPI [24]) when tested on the test set, are given in Table 1. We could not use VirusHostPPI [4] as their model is only available online which precludes us from running an excessive number of predictions required for our test set.

**Table 1:** Model (RF, SMV, MLP) performance metrics without feature selection and comparison with available PPI prediction models.

PPI Prediction Model	TPR	TNR	ACC	F1	MCC	AUC	AUPR
RF^a^ (w/o feature selection)	0.845	0.816	0.818	0.388	0.397	0.904	0.499
SVM^a^ (w/o feature selection)	0.821	0.816	0.816	0.378	0.383	0.894	0.458
MLP^a^ (w/o feature selection)	0.845	0.796	0.799	0.364	0.374	0.892	0.414
DeNovo	0.968	0.052	0.114	0.130	0.023	0.553	0.078
HOPITOR	0.603	0.528	0.533	0.150	0.066	0.607	0.162
InterSPPI-HVPPI	0.897	0.956	0.952	0.718	0.710	0.978	0.897

^a^Abbreviations: RF: random forest, SVM: support vector machine, MLP: multi-layer perceptron

As tabulated results indicate, our baseline RF model performed slightly better than SVM and both had a better performance compared to MLP based on AUC and AUPR metrics. As our intention here is not to prefer a specific PPI prediction model over the other models but to compare the influence of our feature selection approach across the machine-learning algorithms, we did not opt for a single model. Instead, we assessed the impact on all models. When compared to existing PPI prediction tools, while InterSPPI-HVPPI seems to be the most successful predictor, this may partly be attributable to our dataset creation which is derived from Inter-SPPI-HVPPI’s compilation. Our predictors without feature selection, regardless of the algorithm used, displayed a comparable, if not better, prediction performance.

Using the selected set of features based on various correlation coefficient thresholds, we developed 42 different PPI prediction models using the same machine-learning algorithms (RF, SVM, MLP) and 3 different correlation coefficient metrics (PS, SM, KT) at different threshold levels. We evaluated the impact of feature selection in virus-host PPI prediction in comparison to the baseline model (i.e. without feature selection). The respective performance results are listed in S2 Table.

Out of all models we have constructed, the best performers based on the AUPR with a substantially reduced number of features are listed in Table 2.

**Table 2:** Selected performances of virus–host PPI prediction models with and without (grey highlighted) feature selection.

Model	Host Feature #	Virus Feature #	TPR	TNR	ACC	F1	MCC	AUC	AUPR
RF^a^ (w/o feature selection)	343	343	0.845	0.816	0.818	0.388	0.397	0.904	0.499
RF (PS^a^, threshold = 0.05)	95	88	0.840	0.824	0.825	0.396	0.403	0.904	0.496
RF (SM^a^, threshold = 0.05)	109	120	0.835	0.834	0.834	0.406	0.412	0.907	0.502
RF (KT^a^, threshold = 0.04)	86	95	0.835	0.825	0.826	0.395	0.402	0.903	0.497
SVM^a^ (w/o feature selection)	343	343	0.821	0.816	0.816	0.378	0.383	0.894	0.458
SVM (PS, threshold = 0.04)	195	195	0.821	0.814	0.814	0.376	0.380	0.891	0.453
SVM (SM, threshold = 0.04)	311	309	0.825	0.811	0.817	0.377	0.380	0.891	0.448
SVM (KT, threshold = 0.03)	243	246	0.820	0.810	0.811	0.371	0.376	0.892	0.446
MLP^a^ (w/o feature selection)	343	343	0.845	0.796	0.799	0.364	0.374	0.892	0.414
MLP (PS, threshold = 0.03)	195	195	0.831	0.779	0.778	0.346	0.360	0.892	0.406
MLP (SM, threshold = 0.03)	311	309	0.838	0.805	0.807	0.372	0.380	0.891	0.419
MLP (KT, threshold = 0.03)	243	246	0.812	0.811	0.811	0.369	0.372	0.883	0.408

^a^Abbreviations: RF: random forest, SVM: support vector machine, MLP: multi-layer perceptron; PS: Pearson, SM: Spearman’s rank, KT: Kendall’s τ

**Fig 3:**
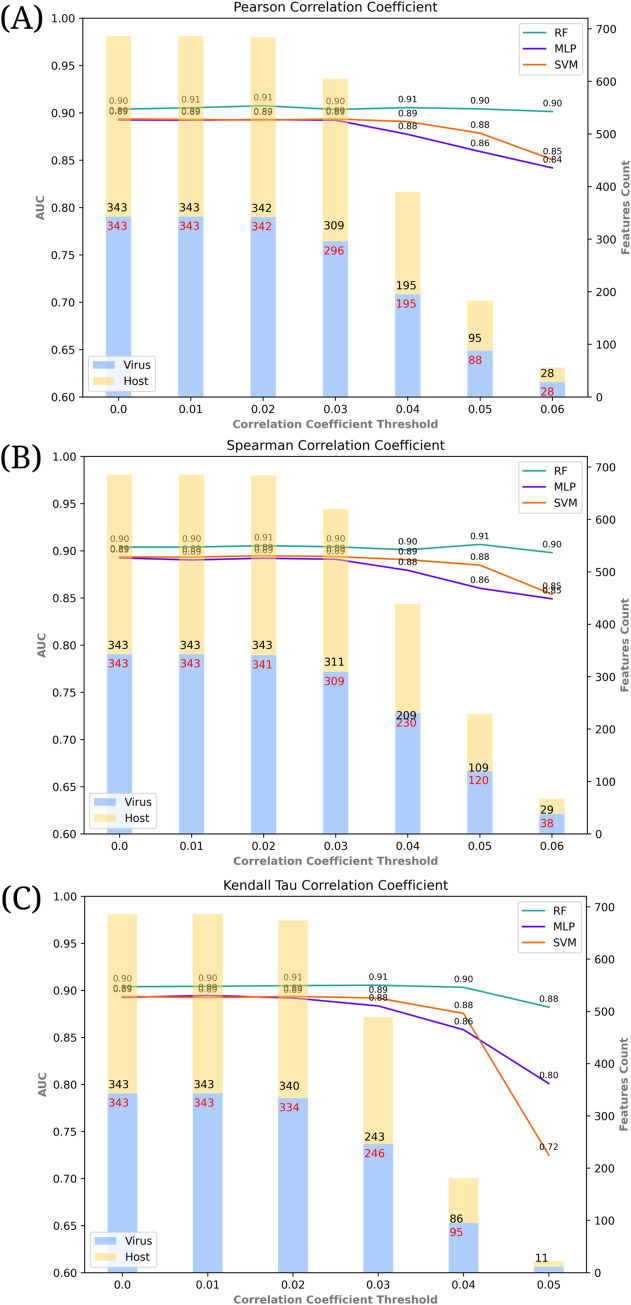
Impact of feature selection on PPI prediction models based on (a) Pearson (b) Spearman and (c) Kendall Tau correlation coefficient. X-axis shows different thresholds used for correlation coefficients. Bars indicate the number of virus (blue) and host (yellow) features. Y-axis on the left shows the Area Under Curve and the one on the right side shows the number of features. Lines indicate the RF (green), MLP (purple), and SVM (orange) model.

Fig 3 provides a visual comparison of the impact of several correlation coefficient metrics along with the resulting feature vector sizes (number of selected virus and host tripeptides) on the performances of PPI prediction models.

As mentioned in the Section Dataset Preparation, the test dataset used in this study is heavily imbalanced with a far higher number of negative pairs (114,563) compared to positive pairs (8,375). The performance metrics MCC, F1, and AUPR, though seemingly low, are actually not necessarily a sign of unfavourable performance. In terms of the metric AUPR, the baseline AUPR for a test dataset of size 122,938 with 8,375 positives corresponds to 0.068 while the AUPR of the RF models is around 0.5; an obviously better value. Likewise, reported F1 values rely on the positive class label which refers to the minority class. In this particular case, unlike intrahost PPI prediction, the F1 value in negative class prediction (i.e. virus protein and host protein are not interacting) is as important as the F1 value for the positive class because this is indicative of the potential that the virus is not capable of infecting a host. So, a weighted F1 as an alternative could have been used for this domain which has a value of >0.85 for our models (data not shown). Finally, although MCC is reported to be unsuitable for classification accuracy measurement on imbalanced datasets, as assessed in similar studies by Zhu [38], we reported it as standard practice employed widely in applications of machine learning methodologies.

Overall, the performance of PPI prediction models was not compromised by feature selection. In particular, RF model with the PS correlation coefficient seems to provide a greater feature reduction ratio from 686 to 56 features (%91.8 at threshold level 0.06) while sustaining the prediction performance in terms of both AUC (reduced by 0.003) and AUPR (reduced by 0.007). In other words, using less than 10% of the originally extracted feature set, we achieved almost similar prediction performance. Of note, the lower threshold values we observed during this study are likely arising from the high diversity of taxa in our PPI dataset.

### Investigation of selected tripeptide features in virus-host PPI structural context

Fisher’s exact test results indicate a significantly higher proportion of intersection (i.e. tripeptides existing both in correlation and structure-based selection) for PS, at all thresholds and for SM except for threshold 0.06. But for the KT correlation coefficient, although the proportion of intersection is higher at thresholds 0.03 and 0.04, the differences are statistically not significant. We provided all Fisher’s exact test details in S4 Table. While PS demonstrates the best results, overall, the results suggest that the choice of correlation coefficient metric has an effect on the detection of the host-virus tripeptides with relevant structural context (i.e. potential contact tripeptides). The significance of structurally-relevant selection is encouraging regarding the use of the correlation coefficient-based feature selection.

To implement an exemplary qualitative analysis, we picked the PDB entry (PDB ID: 4YSI) with the highest resolution. On the human side tripeptides SNF, FMA, NFM, MAW, AWS, WSE, SEV, and on the KSHV side tripeptides EGP, PSG, PGE, SPG, GEG, GPS were identified as the contact tripeptides. Altogether, host tripeptides resulted in 7 distinct tripeptides and likewise, viral tripeptides resulted in 6 distinct tripeptides when encoded in a reduced alphabet.

We compared whether these contact tripeptides exist among the correlation coefficient-based selected tripeptides. At least one component of both the viral and host contact tripeptide sets was co-occurring in our selected tripeptides up until the threshold of 0.04 for PS, 0.05 for SM, and 0.03 for KT correlation coefficient, respectively.

In Table 3 we have presented the intersection of tripeptides occurring both in the contact tripeptide set and in the correlation coefficient-based selected tripeptide set.

**Table 3:** Contact tripeptides selected by correlation coefficient-based approach.

C.C.^a^ Metric	C.C. Threshold	Host Tripeptides	Virus Tripeptides
PS^**a**^	0.0	SNF-FMA-NFM-MAW-AWS-WSE-SEV	EGP-PSG-PGE-SPG-GEG-GPS
PS	0.01	SNF-FMA-NFM-MAW-AWS-WSE-SEV	EGP-PSG-PGE-SPG-GEG-GPS
PS	0.02	SNF-FMA-NFM-MAW-AWS-WSE-SEV	EGP-PSG-PGE-SPG-GEG-GPS
PS	0.03	SNF-FMA-NFM-MAW-AWS-WSE-SEV	EGP-PSG-PGE-SPG-GEG-GPS
PS	0.04	SNF-AWS-WSE-NFM-SEV	EGP-PSG-PGE-SPG-GPS
SM^**a**^	0.0	SNF-FMA-NFM-MAW-AWS-WSE-SEV	EGP-PSG-PGE-SPG-GEG-GPS
SM	0.01	SNF-FMA-NFM-MAW-AWS-WSE-SEV	EGP-PSG-PGE-SPG-GEG-GPS
SM	0.02	SNF-FMA-NFM-MAW-AWS-WSE-SEV	EGP-PSG-PGE-SPG-GEG-GPS
SM	0.03	SNF-FMA-NFM-MAW-AWS-WSE-SEV	EGP-PSG-PGE-SPG-GEG-GPS
SM	0.04	SNF-AWS-WSE-NFM-SEV	PSG-PGE-SPG-GEG-GPS
SM	0.05	WSE-SEV	PGE
KT^**a**^	0.0	SNF-FMA-NFM-MAW-AWS-WSE-SEV	EGP-PSG-PGE-SPG-GEG-GPS
KT	0.01	SNF-FMA-NFM-MAW-AWS-WSE-SEV	EGP-PSG-PGE-SPG-GEG-GPS
KT	0.02	SNF-FMA-NFM-MAW-AWS-WSE-SEV	EGP-PSG-PGE-SPG-GEG-GPS
KT	0.03	SNF-AWS-WSE-NFM-SEV	PSG-PGE-SPG-GEG-GPS

^a^Abbreviations: C.C.: Correlation Coefficient; PS: Pearson, SM: Spearman’s rank, KT: Kendall’s τ.

Independent of the correlation coefficient metric, in the majority of the cases the contact tripeptides are selected in decreasing numbers by the threshold, as expected. The capability of picking up the same tripeptides by our correlation-based selection as those calculated through protein structure data is promising.

The intersection between tripeptides selected through our correlation coefficient-based approach and distance calculation based on protein structures suggests the co-evolution of respective peptides in virus and host can potentially be identified using correlation metrics, though with low thresholds, even in a high level of taxa diversity.

We anticipate that the higher the availability of high-quality virus and host protein complex structures reaches, the more means we will have to validate this hypothesis.

## Conclusions

In this study, we presented a correlation-coefficient-based approach to select tripeptide features extracted from interacting virus and host proteins; a crucial preliminary step for the creation of virus-host PPI prediction tools. We demonstrated that our approach is able to substantially decrease the feature space without sacrificing the predictive power. PS -regardless of the machine-learning algorithm used in the virus-host PPI prediction model- provides the best performance with respect to the baseline model. In particular, the performance of RF model with PS correlation coefficient (threshold 0.05) as measured by AUPR dropped by 0.003 and AUC stayed the same while achieving a 73.3% (from 686 to 183) reduction in the number of tripeptide features.

We also explored potential biological foundations of feature selection by investigating the structural context of selected tripeptides. Correlation-coefficient-based feature selection methodology gave promising results. Qualitatively, it enables the selection of individual contact tripeptides as features. Quantitatively, it favors the selection of a significantly higher number of structurally relevant (contact) tripeptides. We also believe correlation-coefficient-based feature selection may be revealing potential co-evolution patterns among virus-host proteins.

In general, this correlation-coefficient-based feature selection approach can be used for any virus-host PPI prediction tool relying on a tripeptide (or any n-peptide) frequency-based feature extraction scheme. Hence, we believe our approach will bring new perspectives to help the development of new or improvement of existing virus-host PPI prediction tools.

## Supporting information

S1 Table 5-fold cross validation results using stratified viral family selection. (XLSX)

S2 Table Performance results showing the impact of feature selection in virus-host PPI prediction. The impact of feature selection in virus-host PPI prediction is evaluated in comparison to the baseline model (i.e. without feature selection).
(XLSX)

S3 Table The complete list of PDB entries. (XLSX)

S4 Table All Fisher’s exact test details. (XLSX)
